# Ab Initio Density Functional Theory Calculation: Americium Hydrolysis Mechanism

**DOI:** 10.3390/ma17030572

**Published:** 2024-01-25

**Authors:** Na Shan, Tao Gao

**Affiliations:** Institute of Atomic and Molecular Physics, Sichuan University, Chengdu 610065, China; 2022326060001@stu.scu.edu.cn

**Keywords:** reaction mechanism, ab initio, density functional theory, topology analysis, microscopic reaction mechanism

## Abstract

The hydrolysis mechanism of americium was calculated using density functional theory, and the detailed microscopic reaction mechanism was obtained. The results show that americium reacts with water along the octet state to produce oxides and H_2_, and that this reaction is exothermic. The interaction between Am and O atoms gradually changes from initially electrostatic interaction to covalent interaction, and continues to strengthen. During the reaction process, Am atoms always lose electrons, the 5f orbital is obviously involved, and there is df orbital hybridization. This study provides the necessary theoretical data support for the theoretical and experimental study of the actinide system.

## 1. Introduction

As the main materials of the nuclear industry, actinides have been widely studied, including their microelectronic structure, molecular reaction properties, surface interactions, and so on; however, the main research has been focused on light actinides (Th–Pu) and the lighter lanthanide element (Ce) [1,2,3,4,5,6,7,8,9,10,11]. It is well known that actinides contain 5f electrons, which determines their unique properties. In the book *Chemistry of Actinides and Transactinides* [12], it was pointed out that a major electronic transition occurs between the light actinides and the heavy actinides, from Th to Pu; with each additional 5f electron, it enters the conduction band, making the chemical bonds and interactions stronger, and the atoms closer together, thereby reducing the atomic volume. Am is the No. 95 element in the f region of the periodic table, which is a radioactive subactinide element that is obtained artificially and has active chemical properties. Americium was first identified in 1944 by Seaborg et al. in the metallurgical laboratory of the University of Chicago, in plutonium irradiated by reactors [13], and is a major source of long-term radioactive toxicity in nuclear waste.


Pu239n,γPu240n,γPu241→−β−Am241

In the light actinide metals (Ac–Pu), the 5f electrons are known to be mobile rather than localized [14]. The outermost electron configuration of americium is [Rn]5f^7^7s^2^, and the ground-state 5f orbital electrons are half-full; these electrons are localized at each lattice site, and are chemically inactive. Due to its unique position and properties in the actinides, americium has proven to be a material of great interest to both theorists and experimentalists over the past decades [15]. Studies have shown that Am has a variety of isotopes [16], which are widely used in radioactive sources such as isotope thickness gauges and isotope X-ray fluorescence meters, smoke CBE detectors, thermometers, automatic fire alarm instruments, and medicine. It is closely related to our lives, and it is also a radioactive element that needs to be focused on in the treatment of “nuclear waste” [17]. In a recent *Nature* report, a briefing on nuclear waste powering spacecraft caught our attention [18], with researchers saying that using nuclear waste to provide power systems could play a role in missions without solar power. The report said that a device powered by the radioactive isotope Am241 could replace existing plutonium-238 batteries. The core of the cell is a fuel ball made of Am241, and the researchers showed that Am241 can be extracted from the reprocessed nuclear fuel used in civilian power plants to make a fuel ball that forms the core of the cell. However, what is the microchemical mechanism during the application? What way will the surrounding environment affect it? Will it affect the use of the fuel ball? To uncover answers to these questions, a detailed discussion of its basic nature is required.

Currently, the research on americium’s chemical properties and reaction behavior is still in its infancy, and more experimental and theoretical studies are needed to reveal its chemical characteristics. Experimental difficulties and the lack of theoretical data have made it urgent to investigate the reactivity of americium. Based on these factors, this study is based on the atomic molecular level of americium, and analyzes the microscopic properties of the hydrolysis process that can more directly explain the 5f electron contribution and the series element properties with the atomic number trend for the spent fuel after processing; radioactive pollution control and radioactive isotope decomposition purification; and more effectively provide the theory of the elements and fill the relevant data of the elements. This is of great significance to the development of new space power systems. The chemical properties, the basic principles of the hydrolysis, and the reaction mechanism will be described in detail in this paper.

## 2. Method

The calculation process uses density functional theory combined with transition state theory. Through different calculation methods, the relativistic effective atomic real potential SDD with 60 central electrons is used for Am, and the 6-311++G(d,p) basis set is used for H and O atoms. As a quantum mechanical method to study the electronic structure of multi-electron systems, DFT is widely used in the study of atomic and molecular properties, computational material chemistry, and so on. In DFT, electron density is introduced to describe the physical quantity of the system, and has been widely used in various fields of material calculation, and in atomic and molecular physical and chemical properties research. It has the advantages of short computation times, fast convergence, and accurate results. The above methods were proven to be applicable to related research [8,9,19,20,21,22]. All of the calculations for this study were carried out through the G09 program [23].

The initial reactant structure was first designed, and the intermediate and transition states were searched using frequency analysis, where the intermediate has no imaginary frequency and the transition state structure has one and only one imaginary frequency. The intrinsic reaction coordinate (IRC) [24,25] calculation of the transition state structure ensures that the transition state obtained in the study can correctly link the initial and final states and reflect the interaction process completely. Without considering the symmetry, the structure optimization of each special point was carried out, and the relative energies obtained include the zero-point vibration function (ZPVE). Finally, the potential energy profile of the minimum energy path of the reaction process was obtained. The expected value of <S^2^> is taken into account in the calculation to ensure minimal spin contamination.

In addition, the electronic structure evolution data of the reaction path were calculated, and the electronic localization function (ELF) [26,27,28] and atom in molecule (AIM) [29] were calculated to investigate the evolution of the bond and the properties of bond critical points (BCP) in the electron density gradient field. In order to understand the role of the 5f orbital in the reaction process, we calculated the total and partial state densities (TDOS and PDOS) and the overlapping state densities (OPDOS) [30] of the system. At the same time, natural population analysis (NPA) was used to obtain the charge change at each stationary point and the atomic orbital contribution. The above calculations were carried out using Multiwfn3.8 [31].

## 3. Results and Discussion

### 3.1. Ground-State Structures and Energy

When studying the microscopic reaction mechanism of atoms and molecules, the discussion of spin multiplicity is very important, which is helpful to determine the ground state of the reaction process. The calculation method adopted in this study has been proven to be reliable through a large number of studies. In order to improve the accuracy of the calculation, the relative energies of the ground state and low excited state of Am atoms were first calculated and compared with the experimental values, as shown in Table 1. The results show that the calculated results of the above methods are reasonable compared with the experimental values. For Am atoms, the ground state is an octet, followed by a dectet and a sextet. After comprehensive consideration, the calculated results of the PW91 method were used for data analysis.

In order to obtain the microscopic mechanism of the reaction between americium atoms and water molecules, the initial and final states, and the metastable and transition states were first designed and optimized; the doublet, quartet, sextet, and octet were also considered, which is helpful to obtain the reaction process of the system.

**Figure 1:**
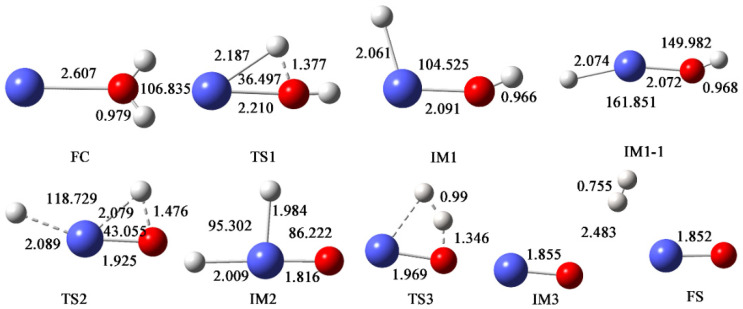
The geometric structures and parameters of each special point of the lowest reaction path on the Am + H_2_O potential energy surface (PES) calculated using PW91/SDD. Bond distances are in Å, and angles are in degrees.

**Figure 2:**
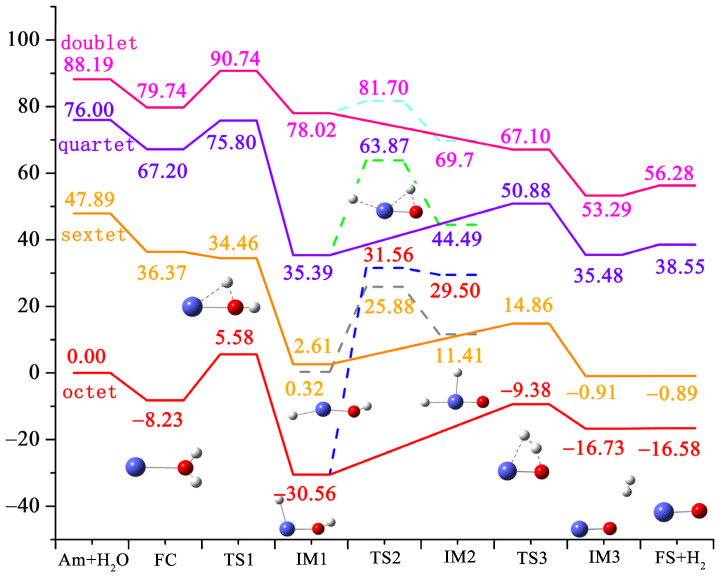
Potential energy surface of the reaction process calculated using PW91/SDD.

Figure 1 shows the dissociation process of hydrogen and oxygen atoms in water molecules, and the trajectories of atomic motion during the reaction; the parameter information of each structure is marked. Figure 2 shows the potential energy profile of the reaction process, reflecting two possible paths of the whole process, namely the H_2_ dissociation path and the isomerization path, corresponding to solid and dashed lines, respectively. Each stationary point structure corresponds one-to-one to the structure in Figure 1. (FC means reactant complex, TS means transition state, IM means intermediate species, FS means final state).

Next, the reaction process is explained in detail. In this study, four different spin multiplicities were calculated, and the results show that the eight-fold state is the lowest energy reaction path. The sum of the initial energies of the two reactants was used as the starting point. Firstly, americium atom and water molecules come close to each other through electrostatic interaction to form the initial complex FC, which has an energy of −8.23 kcal/mol relative to the initial zero point; that is, the process of generating the initial complex is exothermic. After the formation of FC, the O-H bond in the water molecule begins to break, which requires “crossing” 13.81 kcal/mol of energy to generate the first metastable state IM1 after the transition state TS1. At this time, a hydrogen atom in the water molecule completely dissociates and moves to the americium atom in the molecular plane. The structure is −30.56 kcal/mol relative to the initial zero point, indicating that the transition from TS1 to IM1 is a sufficiently expulsive process. From IM1 onwards, the reaction proceeds in two directions. The first is the H_2_ dissociation path, in which the other hydrogen atom in the water molecule dissociates from the oxygen atom and moves towards the first hydrogen atom. This process needs to overcome 21.18 kcal/mol energy to reach TS3, and the relative energy of this structure is −9.38 kcal/mol. As the two hydrogen atoms gradually approach, the metastable state IM3 is obtained. At this time, the distance between the two H atoms is 0.755, which is close to the bond length of hydrogen molecules. The other molecular fragment is AmO, with a bond length of 1.855 Å. The relative energy of IM3 is −16.73 kcal/mol, which is also an exothermic process. The resulting total energy of AmO and H_2_ is −16.58 kcal/mol. The second path is the isomerization path. After IM1, the H atoms in water dissociate from oxygen and move to americium atoms. This process requires up to 62.12 kcal/mol energy, and finally generates H_2_AmO. Therefore, the reaction of americium atoms with water occurs mainly in the dissociation path of H_2_. It should be noted that in this process, the intermediate IM1 first passes through a barrier-free isomerization process to generate IM1-1, corresponding to the angle of H3-Am-O from 104.525° to 161.851°, as shown in Figure 1. The corresponding energy is reduced by 2.29 kcal/mol. After that, metastable IM2 is generated through TS2, with lower energy.

Table 2 shows the relative energy of the lowest energy path of the reaction process, and the value of S^2^ for each structure. It can be found that the influence of spin contamination is very small. For the hexahedral IM1-1, TS2, and IM2 states, the spin contamination is slightly larger because the reactions of these three structures are hexahedral states; thus, there is spin contamination, but it is still within the error range.

### 3.2. Bonding Analysis

**Figure 3:**
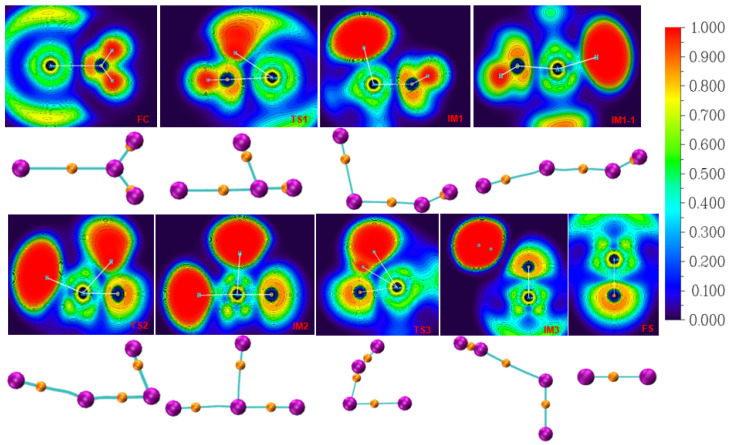
ELF shadow plane projection diagram and AIM topology analysis diagram of metastable and transition states.

Next, the topological properties of the reaction system were analyzed in detail, and the fireworks properties of chemical bonds in the reaction process were studied using the electronic localization function (ELF) and atom-in-molecule (AIM) methods, in order to obtain a deep understanding of the microscopic reaction mechanism. Figure 3 shows the projection of the electron localization function for the reaction of americium with water molecules and atom-in-molecule topology analysis diagram.

The electronic localization function shadow plane projection map can directly reflect the electronic locality of each special point. The electronic localization function (ELF) value is between 0 and 1, and the red represents the area with strong electronic locality; all of the electronic localization function maps are drawn in the XY plane. In the critical points and bond sizes of the (3, −3) and (3, −1) bonds in Figure 3, the purple ball represents the nuclear critical point, the orange ball represents the bond critical point, and the cyan line represents the topological path between the two types of critical points, which becomes the bond path.

Atom-in-molecule data can quantitatively reflect the formation and breakage of chemical bonds, as follows:

(1) $$\nabla^{2}\rho (r)=2G(r)+V(r)$$

(2) H(r)=G(r)+V(r)

where ρ(r) is the electron density, and V(*r*) is the potential energy density, both of which are closely related to the strength of the bond. $\nabla^{2}\rho (r)$ is the Laplacian function of the electron density, while the negative region corresponds to the electron condensation region. *G*(*r*) is the kinetic energy density, and *H*(*r*) is the energy density, which shows the energy of the electron at a certain point; it is the sum of the kinetic energy density and the potential energy density. As for the bonding effect, the H(r) of (3, −1) BCP (bond critical point) proposed by Cremer and Kraka is used as the judgment basis to illustrate the problem. When the η index is less than 1, it is considered to be a closed-shell interaction; when the η index is greater than 1, it is considered to be a covalent interaction, and higher values indicate stronger covalent interactions.

There is no bisynaptic valence basin between Am and O atoms of the initial complex FC. In combination with AIM in Table 3, the ρ(r) and H(r) between the Am-O bonds are very small, and the η index is less than 1, indicating that the two atoms interact with each other in an electrostatic way. The AIM data of the two O-H bonds are consistent, indicating that they exist in the form of water molecules at this time. For TS1, the electron density between Am and O is slightly increased compared with FC, but it is still small. At this time, the red and green areas between O-H3 bonds increase, indicating that the electron localization is reduced, and the corresponding electron density and energy density are also reduced, indicating that after H3 atoms dissociate from O_H2O_ and form IM1, the electron density of H3 atoms is obviously transferred around Am atoms; however, it seems that there is no bond formation, but there is closeness through electrostatic interaction.

For the H_2_ dissociation path, from IM1 to TS3, H4 atoms on the O_H2O_ atoms are moving away from and close to the H3 atom. Combined with AIM data, the electron density between Am-O is also enhanced, while the electron density and energy density between O-H4 are both reduced. After the formation of IM3, the electron density and energy density between O-H4 are close to 0, and the chemical bond is completely broken. However, the red region between H3 and H4 atoms is obviously enhanced, and the corresponding energy density increases; moreover, η > 1, which means that the H_2_ molecule has been formed. Moreover, the synaptic valence basin between the oxygen atom and the two hydrogen atoms completely disappears, indicating that the H_2_ molecular fragment moved away from the Am-O fragment, resulting in AmO and H_2_.

### 3.3. Density of States (DOS)

**Figure 4:**
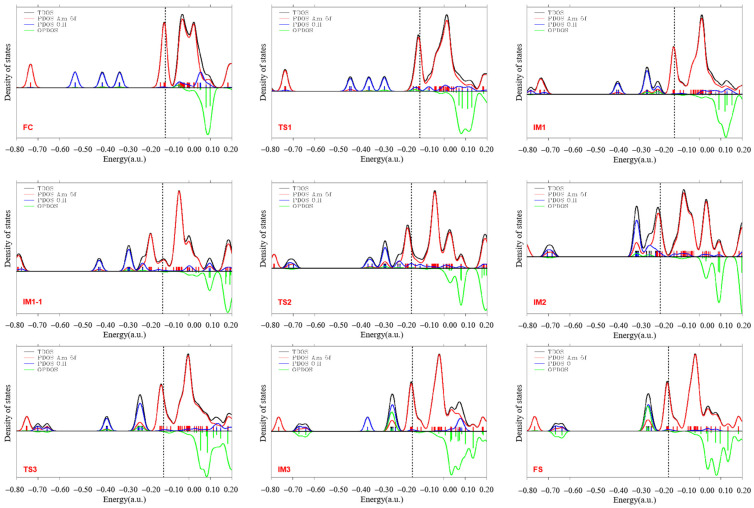
DOS curves for the Am + H_2_O reaction process drawn using the PW91/SDD method.

The 5f electron bonding behavior of the reaction is discussed in terms of DOS. The outermost electron configuration of americium is 5f^7^7s^2^, with a half-full f orbital. In this study, the TDOS, PDOS, and OPDOS were calculated to analyze the orbital composition and orbital contribution in detail. It should be noted that the calculation process for the density of states adopts the basis set that does not contain the dispersion function. Figure 4 shows the molecular orbital density-of-states map for the title reaction, with the left axis representing the values of the TDOS and PDOS, and the right axis representing the values of the OPDOS in a.u. The vertical black dotted line indicates the position of the highest occupied molecular orbital (HOMO). A Gaussian function was used for the broadening function, and the FWHM is 0.03 a.u. OPDOS values greater than or less than 0 represent bonding or antibonding interactions, respectively. When the OPDOS is 0, it means that two fragments have non-key characteristics. The results show that in the region left of −0.2 a.u., the PDOS of the oxygen atom is closer to that of the TDOS, so it is mainly the orbital of the O atom that plays a major role. The PDOS of the 5f orbital of Am in the right region is closer to that of the TDOS, especially in the HOMO position, and the values of the two curves are almost close, indicating that in the HOMO position, it is the 5f orbital of Am that plays a major role. From the DOS diagram of the initial complex FC, it can be seen that the OPDOS values of the 5f electrons of Am and the O and H atoms are all 0, indicating that they have non-bonding interactions, which once again verifies the conclusions obtained before. And as the reaction progresses, the region to the left of the HOMO gradually favors the bonding between the 5f orbital and the O and H atomic bonds.

### 3.4. Natural Population Analysis (NPA)

Finally, the natural population analysis of the title reaction was studied to determine the number of electrons carried by specific atoms and atomic orbitals in the reaction process. The basis set 6-311G(d,p) without the dispersion function was also used in the calculation process. Natural population analysis (NPA) has higher numerical stability, and can better describe the electron distribution in compounds with higher ionic properties. In Table 4, the charge and natural electron configuration of the reaction are listed. The results show that the total charge of each structure is always 0, and the americium atom is always positively charged as an electron donor, while the oxygen atom is negatively charged as an electron acceptor. The electron gains and losses of different atoms are closely related to the electronegativity of the atom, which is 1.13 for Am, 3.44 for O, and 2.20 for H. According to the electronegativity rule, O atoms easily gain electrons from americium. As the reaction progresses, the number of electrons in the 7s orbital of Am gradually decreases, while the numbers of electrons in the 5f and 6d orbitals gradually increase, indicating that some df hybridization has occurred in Am, in which the contribution of 5f electrons is greater.

## 4. Conclusions

In this study, density functional theory (DFT) and ab initio methods were used to predict the microscopic reaction mechanism between americium and water molecules. Different spin multiplicities were considered, and the lowest energy reaction path was obtained. The chemical bond changes and orbital interactions of the reaction paths were determined using various topological methods, which provide an important data reference for the microscopic reactivity study of actinides.

The findings can be summarized as follows: In the two designed reaction paths, the reaction of H_2_ dissociation is carried out along the octet energy surface, and AmO and H_2_ are finally generated, and 16.58 kcal/mol of energy is released. The isomerization path presents a significant energy barrier, so this reaction process does not easily occur. During the reaction, the Am-O interaction is carried out by electrostatic interaction, and the interaction between Am and O atoms increases gradually with the progress of the reaction. The resulting H3-H4 bond satisfies the conditions for the existence of H_2_, and H_2_ is finally produced. During the reaction, the 5f orbital, which is mainly Am, plays a major role in the metastable and transition states. In addition, as the reaction proceeds, Am atoms lose electrons and O atoms gain electrons, and df hybridization also exists in this process.

## Figures and Tables

**Table 1:** Relative energies of the ground state and low excited state of Am atoms (kcal/mol).

	Species	b3lyp	pbe0	pw91	Exptl. ^a^
Am	[Rn]5f^7^7s^2^(6)	50.70	68.61	47.89	42.93
	[Rn]5f^7^7s^2^(8)	0.00	0.00	0.00	0.00
	[Rn]5f^7^6d7s(10)	43.30	35.94	36.23	41.47

^a^ http://www.lac.universite-paris-saclay.fr/Data/Database/Tab-energy/Americium/Am-el-dir.html (accessed on 26 December 2023).

**Table 2:** Relative energies and the expected value of S^2^ of the lowest reaction path calculated using PW91/SDD (kcal/mol).

	Am + H_2_O	FC^8^	TS1^8^	IM1^8^	IM1-1^6^	TS2^6^	IM2^6^	TS3^8^	IM3^8^	FS^8^
E	0.00	−8.23	5.58	−30.56	0.32	25.8	11.41	−9.38	−16.73	−16.58
<S^2^>	-	15.77	15.89	15.77	9.90	9.58	8.98	15.78	15.80	15.80
s(s + 1)	-	15.75	15.75	15.75	8.75	8.75	8.75	15.75	15.75	15.75

**Table 3:** Topological properties of the electron density for (3, −1) BCP.

	Bond	ρ	σ^2^ρ	*G*(*r*)	V(*r*)	*H*(*r*)	η
^8^FC	Am-O	0.034	0.147	0.035	−0.034	0.001	0.163
	O-H3	0.344	−2.280	0.071	−0.711	−0.641	1.696
	O-H4	0.344	−2.281	0.071	−0.711	−0.641	1.696
^8^TS1	Am-O	0.081	0.394	0.108	−0.117	−0.009	0.170
	O-H3	0.124	0.012	0.064	−0.126	−0.062	0.493
	O-H4	0.347	−2.298	0.073	−0.720	−0.647	1.673
^8^IM1	Am-O	0.112	0.499	0.151	−0.178	−0.027	0.180
	Am-H3	0.074	0.064	0.040	−0.064	−0.024	0.373
	O-H4	0.354	−2.316	0.078	−0.734	−0.657	1.666
^6^IM1-1	Am-O	0.118	0.488	0.152	−0.182	−0.030	0.194
	Am-H3	0.076	0.033	0.033	−0.058	−0.025	0.433
	O-H4	0.351	−2.316	0.075	−0.729	−0.654	1.674
^6^ts2	Am-O	0.194	0.483	0.222	−0.323	−0.101	0.294
	Am-H3	0.073	0.044	0.033	−0.056	−0.023	0.424
	O-H4	0.103	0.072	0.057	−0.096	−0.039	0.438
^6^im2	Am-O	0.254	0.476	0.295	−0.471	−0.176	0.333
	Am-H3	0.088	0.016	0.037	−0.069	−0.033	0.506
	Am-H4	0.090	0.031	0.043	−0.078	−0.035	0.448
^8^TS3	Am-O	0.171	0.533	0.212	−0.291	−0.079	0.252
	O-H4	0.130	0.026	0.075	−0.143	−0.068	0.494
	H3-H4	0.143	−0.311	0.025	−0.128	−0.103	0.943
^8^IM3	Am-O	0.228	0.537	0.277	−0.420	−0.143	0.290
	O-H4	0.009	0.026	0.006	−0.005	0.001	0.195
	H3-H4	0.254	−1.006	0.001	−0.253	−0.252	1.197
^8^FS	Am-O	0.231	0.535	0.280	−0.426	−0.146	0.292

ρ(bcp) and ∇^2^ρ(bcp) are in a.u. For H_2_, ρ(bcp) = 0.259 a.u.; ∇^2^ρ(bcp) = −1.021 a.u.

**Table 4:** NPA charge analysis of Am + H_2_O.

	NPA	Am	O	H3	H4
^8^FC	Charge	0.04879	−1.01363	0.48238	0.48246
	Natural Electron Configuration	7S^1.91^5f^6.99^6d^0.04^7p^0.01^	2S^1.78^2p^5.22^3S^0.01^3p^0.01^	1S^0.51^2S^0.01^	1S^0.512^S^0.01^
^8^TS1	Charge	0.65194	−1.15263	0.00025	0.50044
	Natural Electron Configuration	7S^1.20^5f^6.94^6d^0.20^7p^0.02^	2S^1.84^2p^5.30^3p^0.01^	1S^0.99^2S^0.01^	1S^0.49^
^8^IM1	Charge	1.27564	−1.20985	−0.54767	0.48188
	Natural Electron Configuration	7S^0.51^5f^6.81^6d^0.41^7p^0.03^	2S^1.80^2p^5.40^	1S^1.55^	1S^0.51^
^6^IM1-1	Charge	1.07897	−1.14738	−0.42137	0.48979
	Natural Electron Configuration	7S^0.96^5f^6.50^6d^0.43^7p^0.04^8S^0.01^	2S^1.81^2p^5.33^	1S^1.42^	1S^0.51^
^6^TS2	Charge	1.35518	−0.8941	−0.43545	−0.02563
	Natural Electron Configuration	7S^0.44^5f^6.61^6d^0.58^7p^0.06^	2S^1.91^2p^4.97^3p^0.01^	1S^1.43^	1S^1.01^2S^0.01^
^6^IM2	Charge	1.46476	−0.75821	−0.34861	−0.35794
	Natural Electron Configuration	7S^0.32^5f^6.39^6d^0.84^7p^0.08^	2S^1.90^2p^4.85^	1S^1.34^	1S^1.36^
^8^TS3	Charge	1.13815	−1.05612	−0.28414	0.20212
	Natural Electron Configuration	7S^0.47^5f^6.78^6d^0.57^7p^0.06^	2S^1.90^2p^5.15^	1S^1.28^	1S^0.79^2S^0.01^
^8^IM3	Charge	1.04945	−1.03958	−0.03848	0.02862
	Natural Electron Configuration	7S^0.70^5f^6.61^6d^0.62^7p^0.08^	2S^1.93^2p^5.10^	1S^1.04^	1S^0.97^
^8^FS	Charge	1.0308	−1.0308		
	Natural Electron Configuration	7S^0.71^5f^6.60^6d^0.63^7p^0.09^	2S^1.93^2p^5.09^		

## Data Availability

No data were used for the research described in the article.
